# Conservative Management of Retroperitoneal Hematoma Expanded to Prerenal Space Due to Episiotomy in a Woman with Vaginal Delivery, a case report

**DOI:** 10.1016/j.ijscr.2024.109318

**Published:** 2024-01-27

**Authors:** Mohammad Haddadi, Sedigheh Hantoushzadeh, Maryam Deldar Pesikhani, Fatemeh Asadi, Sima Amini, Marjan Ghaemi

**Affiliations:** Vali-E-Asr Reproductive Health Research Center, Family Health Research Institute, Tehran University of Medical Sciences, Tehran, Iran

**Keywords:** Hematoma, Episiotomy, Case report, Vaginal delivery

## Abstract

**Introduction:**

Episiotomy is a procedure during vaginal delivery to facilitate a safer delivery. However, it can also have complications including hemorrhage, perineal tears, infections, and vaginal hematoma which should be managed and monitored carefully.

**Presentation of case:**

A 27-year-old woman with term pregnancy, had a normal vaginal delivery at 39 weeks of gestation, and a large episiotomy was performed due to the estimated neonate weight to prevent shoulder dystocia. She was complicated with a huge pelvic hematoma that was expanded to prerenal space.

**Discussion:**

This complication was managed by conservative therapy, including antibiotic therapy, intensive observation of the patient's situation, and follow-up with a CT scan after consulting with a radiologist. The huge hematoma was reduced.

**Conclusion:**

Noninvasive management and close monitoring for pelvic hematoma due to episiotomy in a low-risk patient are successful; however, consulting with radiologists and experts and a multidisciplinary approach should be considered.

## Highlights

• Episiotomy is a controversial issue among clinicians, and it can cause some complications, including hematoma
• Huge hematoma due to episiotomy should be considered as a diagnosis even for patients without any medical or surgical history.
• Conservative management for hematoma due to episiotomy in a patient without any risk factor is practical; however, it is important to consult with other experts and other therapies should be considered,

## 1 Introduction

Episiotomy is a surgical procedure to incise the vagina and perineum in order to artificially enlarge the introitus and facilitate delivery at the end of the second stage of labor [1]. The episiotomy rate significantly differs between countries due to their policies and practices; for example, it is approximately 4 % in Denmark, and 45 % in Saudi Arabia [2]. Also, A study in Iran showed that the episiotomy rate could be nearly 41.5 % [3]. The World Health Organization (WHO) recommended that the rate of episiotomy should not be more than 10 % [4] because episiotomy has several complications such as hemorrhage, perineal tears, infections, vaginal hematoma, gaping wound, urinary retention, passing stools inability, etc. [5]. Hematoma in the episiotomy site is another uncommon complication due to episiotomy [6,7]. Also, it could be more common and expanded in patients with underlying diseases, such as coagulation disorders [8].

This case presented a retroperitoneal hematoma due to episiotomy in a primigravid woman without any medical history and its management. We used the SCARE guideline to report this patient [9].

## 2 Case presentation

A 27-year-old primigravid woman, at 39 weeks of gestational age, was admitted to the hospital with labor pain. She did not declare any past medical or surgical history, and she had been visited by an obstetrician regularly with suitable prenatal care. She did not have any complications due to pregnancy. The first and second fetal screenings to detect fetal abnormalities were normal. She had used folic acids during her pregnancy.

In the first vaginal examination, her cervix was 40 % effaced and 5 cm dilated with cephalic presentation.

The delivery progressed with regular contractions without any drug induction (1 and a half hours), and episiotomy was performed due to the estimated neonate weight to prevent shoulder dystocia. The newborn was female, 3900 g, with an Apgar score of 9 in 5 min and 10 in 10 min. Her examinations and reflexes were normal.

The placenta and membranes were removed entirely, the uterus contracted, postpartum bleeding was normal, and the internal and external sphincter was intact. Due to the patient's intolerance despite sedation, he was transferred to the operating room to repair the episiotomy. There was no evidence of vaginal or rectal hematoma. The patient was transferred to the recovery room in a stable general condition.

Her hemoglobin was 12.1 g/dL after 6 h of delivery, and her vital signs and general condition were typical. After 12 h, the woman had tachycardia (120 beats/min) and urinary retention; During the vaginal and rectal examination, a hematoma of about 7 cm was felt on the right wall of the vagina, which drained about 200 cc by itself after the test. After that, the patient's urinary retention improved.

**Fig. 1:**
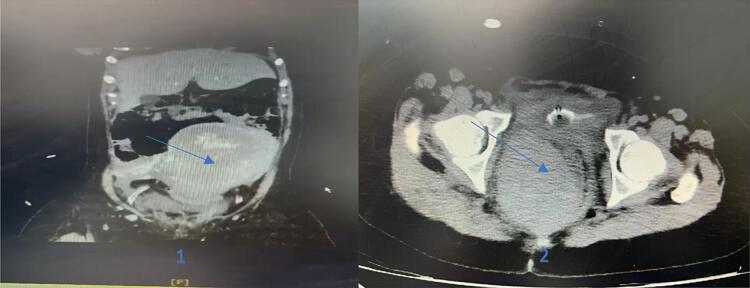
The abdomino-pelvic computed tomography (CT) scan of a large hematoma. 1) The coronal view, 2) the axial view, at right perirectal and presacral spaces extending upward to the pelvic inlet and lower pararenal space. The uterus and rectum were displaced to the left and anterior.

Therefore, she underwent abdominal ultrasonography. Its result showed a hypo-hetero-echoic area in the ischio-rectal area with dimensions of 71 × 108 × 74 mm and a volume of approximately 300 cc, suggesting a hematoma. After that, an abdomen-pelvic CT with intravenous (IV) contrast was requested, and the results showed evidence of a large retroperitoneal hematoma on the side of the peri-sacral space with superior extension to the level of the pararenal space, which had a size of 115 × 75 mm on the right side of the pelvis. Mild fluid was seen in the pelvis, and there were no signs of obstruction or other pathological findings (Fig. 1). Moreover, a laboratory test was requested, and her hemoglobin level was 8 g/dL, so 2 units of packed red blood cells were infused for her.

Interventional radiology consultation was requested, and conservative management was recommended. She underwent antibiotic therapy with clindamycin 900 mg/ IV/ q6hours and ampicillin 1 g/IV/q6hours for 4 days due to fever, but suddenly she had tachycardia (110 beast/min) and fever (39^o^ Celsius). The US was requested for her. It showed a hypo-hetero-echoic area with 66 × 45 × 96 mm and a volume of approximately 140 cc posterior and right lateral of the vaginal canal and rectum, suggesting a reduced hematoma compared to the previous one. Her hemoglobin was 9.2 g/dL. Gentamycin 450 mg/IV/q6hours was added to her treatment, and she underwent antibiotic therapy with 3 medications for another 4 days.

After that, her vital signs were stable (heart rate: 80 beats/min, blood pressure: 100/70 mmHg, respiratory rate: 16, SO2:98 %) and her hemoglobin reached 10.2 g/dL. After stopping the medication, the patient was discharged without fever. The general condition was good and she started breastfeeding. The vaginal discharge was normal, and the episiotomy site was clear without infection.

The patient was visited after a week. Her vital signs were normal, and she had no problem or pain.

## 3 Discussion

This case presented a primigravid woman with no medical and surgical history with a huge pelvic hematoma due to episiotomy after her normal vaginal delivery. Also, she underwent antibiotic therapy due to her fever. No intervention was performed for her hematoma, which recovered after antibiotic therapy.

Episiotomy is a controversial procedure among clinicians, and it could affect maternal-fetal complications and changes during and after delivery, including bleeding, perineal infection, neonatal trauma, and changes in pelvic floor muscles [10,11]. However, some complications from episiotomy can be reduced by the procedure methods and materials [[12], [13], [14]].

Episiotomy is reported as a risk factor for levator hematoma [15]. Also, it can be a risk factor for puerperal hematomas, especially among multigravida women [16]. Although puerperal hematomas are rare, they can be life-threatening for women mainly due to site infections, and appropriate surgical technique and repairing can reduce this complication [17]. In addition, many management could be considered for the patients due to the situation, including conservative therapy, surgical therapy, and endovascular interventions [18]. Maroyi et al. reported a case of retroperitoneal hematoma following vaginal delivery that was managed by laparotomy [19]. Management of retroperitoneal hematoma is very challenging in obstetric wards, and it requires multidisciplinary consulting [20].

In this case, we used antibiotic therapy due to her fever and supportive care after consulting with an expert radiologist despite the large size of the hematoma. Clindamycin plus Ampicillin were our first medications for patients due to recent guidelines and studies, and we added Gentamycin due to her persistent fever [21]. So, this case could show the successful conservative management of pelvic massive hematoma due to episiotomy based on the patient's situation. However, carefully monitored conservative management is an option that should be considered by the clinician alongside other possible methods of treating this rare complication.

## 4 Conclusion

In conclusion, episiotomy is a controversial issue among clinicians, and it can cause some complications, including hematoma. However, it is essential for some women based on their situations, and appropriate techniques during procedures can reduce them. Conservative management for hematoma due to episiotomy in a patient without any risk factor is successful; however, consulting with radiologists and experts and considering other therapies should be on the desk.

## Consent

Written informed consent was obtained from the patient for publication and any accompanying images. A copy of the written consent is available for review by the Editor-in-Chief of this journal on request.

## Ethical approval

Ethical approval for this study was provided by the Ethical Committee of Tehran University of Medical Science on 1 August 2023.

Reference code: 55.tums.853.

## Funding

There is no financial support.

## Author contribution

M.G. and S.H.: conceptualizing

F.A., S.A.: preparing data and pictures

M.H: writing-original draft

M.H., M.G., and M.D.P: Review and edit

All authors reviewed and approved the final manuscript.

## Guarantor

Marjan Ghaemi.

## Research registration number

N/A.

## Conflict of interest statement

The authors declare no conflict of interest.

## Data Availability

Data is available upon request.
